# The synchronized trial on expectant mothers with depressive symptoms by omega-3 PUFAs (SYNCHRO): Study protocol for a randomized controlled trial

**DOI:** 10.1186/s12888-016-1031-2

**Published:** 2016-09-15

**Authors:** Daisuke Nishi, Kuan-Pin Su, Kentaro Usuda, Yi-Ju Jill Chiang, Tai-Wei Guu, Kei Hamazaki, Naoki Nakaya, Toshimasa Sone, Yo Sano, Yoshiyuki Tachibana, Hiroe Ito, Keiich Isaka, Kenji Hashimoto, Tomohito Hamazaki, Yutaka J Matsuoka

**Affiliations:** 1 Department of Obstetrics and Gynecology, Tokyo Medical University, 6-7-1 Nishi-shinjuku, Shinjuku-ku, Tokyo 160-0023 Japan; 2 National Institute of Mental Health, National Center of Neurology and Psychiatry, 4-1-1 Ogawahigashicho, Kodaira, Tokyo 187-8553 Japan; 3 Department of Public Mental Health Policy, Graduate School of Medicine, The University of Tokyo, 4-1-1, Ogawahigashicyo, Kodaira, Tokyo 187-8553 Japan; 4 Department of Psychiatry & Mind-Body Interface Laboratory (MBI-Lab), China Medical University Hospital, No. 2, Yuh-Der Road, Taichung, 404 Taiwan; 5 Graduate Institute of Neural and Cognitive Sciences, College of Medicine, China Medical University, No.91, Hsueh-Shih Road, Taichung, 404 Taiwan; 6 Department of Public Health, Faculty of Medicine, University of Toyama, 2630 Sugitani, Toyama, Toyama 930-0194 Japan; 7 Department of Preventive Medicine and Epidemiology, Tohoku Medical Megabank Organization, Tohoku University, 2-1 Seiryo-machi, Aoba-ku, Sendai, Miyagi 980-8573 Japan; 8 Department of Rehabilitation, Faculty of Health Science, Tohoku Fukushi University, 6-149-1 Kunimigaoka, Aoba-ku, Sendai, Miyagi 989-3201 Japan; 9 Toda Chuo Women’s Hospital, 2-26-3 Kamitoda, Toda, Saitama 335-0022 Japan; 10 Division of Infant and Toddler Mental Health, Department of Psychosocial Medicine, National Center for Child Health and Development, 2-10-1 Ookura, Setagaya-ku, Tokyo 157-8535 Japan; 11 Division of Clinical Neuroscience, Chiba University Center for Forensic Mental Health, 1-8-1 Inohana, Chiba, 260-8670 Japan; 12 Department of Medicine, Toyama Jonan Onsen Daini Hospital, 1-13-6, Taromarunishimachi, Toyama, Toyama 939-8271 Japan; 13 Division of Health Care Research, Center for Public Health Sciences, National Cancer Center (YJM), 5-1-1 Tsukiji, Chuo-ku, Tokyo 104-0045 Japan

**Keywords:** Omega-3 polyunsaturated fatty acids, Eicosapentaenoic acid, Depression, Pregnancy, Prevention

## Abstract

**Background:**

Maternal depression can be harmful to both mothers and their children. Omega-3 polyunsaturated fatty acid (PUFA) supplementation has been investigated as an alternative intervention for pregnant women with depressive symptoms because of the supporting evidence from clinical trials in major depression, the safety advantage, and its anti-inflammatory and neuroplasticity effects. This study examines the efficacy of omega-3 PUFA supplementation for pregnant women with depressive symptoms in Taiwan and Japan, to provide evidence available for Asia. The rationale and protocol of this trial are reported here.

**Methods:**

The Synchronized Trial on Expectant Mothers with Depressive Symptoms by Omega-3 PUFAs (SYNCHRO) is a multicenter, double-blind, parallel group, randomized controlled trial. Participants will be randomized to either the omega-3 PUFAs arm (1,200 mg eicosapentaenoic acid and 600 mg docosahexaenoic acid daily) or placebo arm. Primary outcome is total score on the Hamilton Rating Scale for Depression (HAMD) at 12 weeks after the start of the intervention. We will randomize 56 participants to have 90 % power to detect a 4.7-point difference in mean HAMD scores with omega-3 PUFAs compared with placebo. Because seafood consumption varies across countries and this may have a major effect on the efficacy of omega-3 PUFA supplementation, 56 participants will be recruited at each site in Taiwan and Japan, for a total number of 112 participants. Secondary outcomes include depressive symptoms at 1 month after childbirth, diagnosis of major depressive disorder, changes in omega-3 PUFAs concentrations and levels of biomarkers at baseline and at 12 weeks’ follow-up, and standard obstetric outcomes. Data analyses will be by intention to treat. The trial was started in June 2014 and is scheduled to end in February 2018.

**Discussion:**

The trial is expected to provide evidence that can contribute to promoting mental health among mothers and children in Asian populations.

**Trial registration:**

Clinicaltrials.gov: NCT02166424. Registered 15 June 2014; University Hospital Medical Information Network (UMIN) Center: UMIN000017979. Registered 20 May 2015.

**Electronic supplementary material:**

The online version of this article (doi:10.1186/s12888-016-1031-2) contains supplementary material, which is available to authorized users.

## Background

A systematic review reported the prevalence of depression during pregnancy was 7.4 % for the first trimester, 12.8 % for the second, and 12.0 % for the third [1]. A meta-analysis estimated the prevalence of major and minor depression in the range of 6.5 to 12.9 % during the different trimesters of pregnancy and during the first 12 months postpartum [2]. Depression during pregnancy can have harmful effects on both the mother and child. The mother may experience difficulties performing daily activities, fail to seek prenatal care, have a poor diet, use tobacco, alcohol, or other harmful substances, and be at risk of self-harm or suicide [3]; fetal growth rate may be slower; and the child may have temperament or behavioral problems later [4–6].

The established treatment options for depression include antidepressants, cognitive behavioral therapy (CBT), and interpersonal psychotherapy (IPT). However, there are some limitations to each of these options. In terms of antidepressants, guidelines recommend all antidepressant drugs be used with caution during pregnancy and that selective serotonin reuptake inhibitors such as paroxetine be avoided [7, 8]. In fact, it was found in a population screening study of pregnant women attending antenatal clinics that only 11 % of pregnant women with major depressive disorders (MDD) were actually receiving suitable antidepressant medication [9]. Both CBT and IPT are recommended for pregnant women with mild or moderate depression [10] and an RCT has shown that IPT was effective for depression during pregnancy [11], but pregnant women cannot always access CBT or IPT. Identifying a safe alternative treatment strategy for depression during pregnancy is therefore desirable.

Many meta-analyses of RCTs [12–19], although not all [20, 21], support the positive effects of omega-3 polyunsaturated fatty acid (omega-3 PUFA) supplementation on depressive symptoms. The latest evidence supports the efficacy of omega-3 PUFAs rich in eicosapentaenoic acid (EPA) against depression [14, 16–18].

Omega-3 PUFAs are essential nutrients for maintaining physiological function of the mothers and infants during pregnancy. A previous study showed that omega-3 PUFA content in the brain of pregnant rats can be reduced after a single reproductive cycle when they are deprived of sufficient dietary omega-3 PUFAs, a reduction which may affect neuronal function [22]. In addition, another study reported that the brain decreases in volume in pregnant women [23]. It has been suggested that changes in the phospholipid content of the brain’s membranes could reduce brain size [24]. It is known that the composition of maternal phospholipid membranes can be altered during pregnancy by the fetus scavenging essential fatty acids such as omega-3 PUFAs, and this could potentially change brain morphology [23]. A lack of omega-3 PUFAs, then, may have a harmful effect on maternal mental health as well as on neurodevelopment of the fetus and infant. A naturalistic longitudinal study showed that lower seafood intake in pregnant women was associated with an increased risk of their children having suboptimum neurodevelopment [25]. To date, although two RCTs have failed to show the efficacy of omega-3 PUFAs for pregnant women with depression [26, 27], the supplements used contained high levels of docosahexaenoic acid (DHA) rather than EPA.

In a previous RCT conducted in Taiwan, our group showed the efficacy of supplements with a high ratio of EPA (2.2 g daily) to DHA (1.2 g daily) for depression in pregnancy [28]. However, it is not yet clear what the appropriate amount of supplemental omega-3 PUFAs is and it may differ between countries because of national differences in the consumption of fish, which is a rich source of omega-3 PUFAs. Although fish consumption in Taiwan is higher than in most Western countries, it is half that in Japan [29]. The recommended daily intake for pregnant women in Japan is 1.8 g of omega-3 PUFAs [30], and the usual dose of pure EPA as a prescription drug is also set at 1.8 g daily.

Recently, we suggested the potential efficacy of moderate amount of omega-3 PUFAs supplementation for improving depressive symptoms in our open label trial conducted in Japan and Taiwan [31]. The objective of the present randomized controlled trial is to determine the efficacy of a daily dose of 1.8 g of omega-3 PUFAs supplements (1.2 g EPA and 0.6 g DHA) for depressive symptoms in pregnant women in Japan and Taiwan. Examining the efficacy and safety of 1.8 g of omega-3 PUFAs supplementation in different countries is clinically relevant because not only is fish consumption generally higher and the prevalence of depression generally lower in East Asian countries such as Japan and Taiwan compared with Western countries, but also there are considerable differences between East Asian countries [29]. The study is called the Synchronized Trial on Expectant Mothers with Depressive Symptoms by Omega-3 PUFAs (SYNCHRO), and this paper explains the rationale and describes protocol of the trial. An additional file provides the completed SPIRIT 2013 Checklist (see Additional file 1).

## Methods/Design

SYNCHRO is a multicenter, double-blind, parallel group, randomized controlled trial that will allocate participants to an intervention arm to receive omega-3 fatty acid supplementation or a parallel placebo arm in the ratio of 1:1. The trial will run from June 2014 through February 2018, with participant enrollment between June 2014 and August 2017.

The study was registered at clinicaltrials.gov (NCT02166424) on June 15, 2014 and the University Hospital Medical Information Network (UMIN) Center (UMIN000017979) on June 22, 2015.

### Inclusion criteria

Pregnant women who are between 12 and 24 weeks of gestation will be recruited and followed through their pregnancy and up to 4 weeks after childbirth. The inclusion criteria are:

1. Pregnant and aged ≥20 years
2. Japanese conversational ability at the Japan site or Chinese conversational ability at the Taiwan site to ensure participants have sufficient understanding of the trial’s scope and can provide written informed consent
3. Planning to remain in the Tokyo area or Taichung area for 4–6 weeks after childbirth
4. Edinburgh Postnatal Depression Scale (EPDS) score of ≥9
5. In good physical health as judged by an obstetrician

### Exclusion criteria

The presence of any of the following will result in exclusion from the trial.

1. History or current suspicion of psychosis, bipolar I disorder, substance abuse or related disorder, eating disorder, or personality disorder
2. Serious psychiatric symptoms, such as self-harm behavior, or in need of rapid psychiatric treatment
3. Normal birth not expected (e.g., fetal malformation)
4. History of bleeding disorder such as von Willebrand’s Disease
5. Regular treatment with aspirin or warfarin for the past 3 months
6. Smoking habit of ≥40 cigarettes per day
7. Regular treatment with ethyl icosapentate or regular consumption of omega-3 PUFA supplements for the past 3 months
8. Habit of eating fish as a main dish ≥4 times per week

### Procedure

**Fig. 1:**
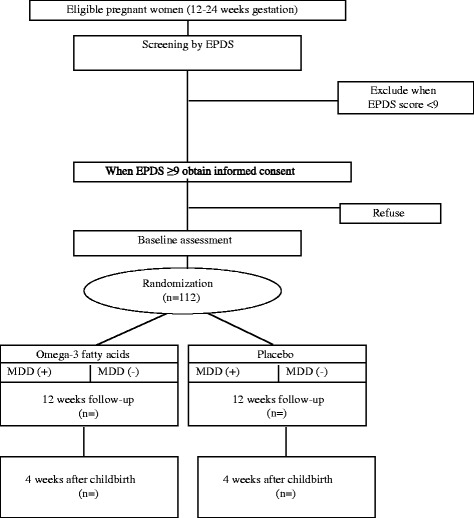
Flow diagram of the study. EPDS: Edinburgh Postnatal Depression Scale; MDD: major depressive disorders

Eligible patients will be screened using the EPDS at three locations in cooperation with obstetricians: Toda Chuo Women’s Hospital and the National Center for Child Health and Development, both in Japan, and China Medical University Hospital, Taiwan. Toda Chuo Women’s Hospital is a local base hospital, and the National Center for Child Health and Development and China Medical University Hospital are academic hospitals. Clinical research coordinators, who will be well-trained nurses, psychologists or psychiatrists, will invite eligible patients to take part in the study. Patients will receive a comprehensive obstetric examination before the study commences and undergo a baseline assessment before randomization. Figure 1 shows a flow diagram of the study protocol.

### Randomization

Central registration involved assigning participants to each group according to an assignment diagram that was developed by a statistician. Using a computer-generated random allocation sequence, they created block randomization tables with two stratification factors: diagnosis of current MDD and study site. The test will be double-blinded and only after all participants have completed the protocol and all the results have been tabulated will the research term be informed of the results of the randomization. Stratification was justified by previous studies: the results of a meta-analysis showed some evidence supporting the benefits of omega-3 PUFAs in individuals diagnosed with MDD but no evidence supporting any benefits in individuals without MDD [32]. Moreover, a previous study showed that seafood consumption predicted prevalence rates of postpartum depression across countries [33].

### Intervention

Participants will take omega-3 PUFAs capsules or placebo capsules for 12 weeks, and can take an entire day’s dosage at once or spread throughout the day. The omega-3 PUFAs capsules have been formulated to each contain 134 mg of EPA and 67.7 mg of DHA, and a total of 9 capsules containing a total of 1206 mg EPA and 609 mg DHA will be administered daily. The placebo capsules have been formulated to contain 320 mg of olive oil and 9.9 mg of omega-3 fatty acids. Trace amounts of semi-deodorized fish oil are added to the control oil so that its smell is not perceivably different from that of the active oil.

Adherence to the regimens will be checked by clinical research coordinators. Whole blood samples (7 ml) collected with ethylene-diamine tetraacetic acid will be taken at baseline, at 12 weeks’ follow-up, and at 1 month after childbirth and omega-3 PUFAs will be determined as described previously [34]. Briefly, erythrocytes will be washed two time with saline and stored at−80 °C until analysis. The method of Bligh and Dyer [35] will be used to extract total lipids, and total phospholipid fractions will be separated by thin-layer chromatography and transmethylated with by treatment with a mixture of HCl and methanol. Next, fatty acid composition will be analyzed by gas chromatography (GC-2014 Shimadzu Corporation, Kyoto, Japan) controlled with GC-solution version 2.3 (Shimadzu Corporation, Kyoto, Japan) and equipped with a DB-225 capillary column (length, 30 m; internal diameter, 0.25 mm; film 0.25 μm; J&M Scientific, Folsom, CA).

### Assessment

The assessment schedule from baseline to 1 month after childbirth is shown in Table 1.

**Table 1:** Outcome measures and assessments

	Baseline	12 weeks	1 month after childbirth
Primary outcome			
HAMD	+	+	+
Secondary outcome			
MINI	+	+	+
EPDS	+	+	+
BDI-II	+	+	+
Plasma	+	+	+
Serum	+	+	+
Other measures			
Demographics	+		
Obstetric outcomes			+
Erythrocyte	+	+	+
Adverse effects		+	+

*HAMD* hamilton rating scale for depression (HAMD), *MINI* mini international neuropsychiatric interview, *EPDS* Edinburgh postnatal depression scale, *BDI*-II beck depression inventory-II

### Outcome measures

#### Primary outcome

The primary outcome is total score on the 17-item Hamilton Rating Scale for Depression (HAMD) [36, 37] at 12 weeks after the start of the intervention. HAMD is a widely used structural interview for assessment of depressive symptoms and has been used during pregnancy and in the postpartum period [26, 28, 38]. Trained psychiatrists, psychologists, or nurses will conduct the structured interviews for the evaluation. To assess inter-rater reliability, 3 well-trained raters in Japan and 2 well-trained raters in Taiwan have independently assessed the same person acting as a patient in a video. The original video was made in Japanese and then translated into Chinese for the raters in Taiwan. The maximum variance in scores among the 5 raters from the expert gold standard score was satisfactory for all the HAMD items. The intraclass correlation coefficient for the total HAMD score among the 5 raters was 0.967 (95 % confidence interval, 0.934–0.986).

#### Secondary outcomes

The secondary outcomes are:

- Total HAMD score at 4 weeks after childbirth
- Major depressive disorder (MDD) as determined by the depression module of the Mini International Neuropsychiatric Interview (MINI) [39] at 12 weeks after starting the intervention and at 4 weeks after childbirth-MINI is a concise structural interview for the major Axis I psychiatric disorders described in the Diagnostic and Statistical Manual of Mental Disorders, Fourth Edition and the International Classification of Diseases and Related Health Problems, Tenth Revision
- Total scores on the EPDS [40] at 12 weeks after starting the intervention and at 4 weeks after childbirth-EPDS is a widely used 10-item self-report scale to assess the severity of perinatal depressive symptoms in the past week; EPDS scores > 9 may indicate the presence of MDD [41]
- Beck Depression Inventory II (BDI-II) [42] at 12 weeks after starting the intervention and at 4 weeks after childbirth-BDI-II is a widely used 21-item self-report scale to measure the severity of depressive symptoms in the past 2 weeks [42]; it was not designed to perinatal mood specifically but has been used during pregnancy and in the postpartum period [28, 43]
- Biomarkers levels at baseline, 12 weeks’ follow-up, and 1 month after childbirth for

- Omega-3 fatty acids concentrations in erythrocytes
- Brain-derived neurotrophic factor (BDNF) in serum
- Estrogen in plasma
- Oxytocin in plasma
- Progesterone in plasma
- Human chorionic gonadotropin in plasma
- Phospholipase A2 in plasma

For the measurement of biomarkers, plasma and serum will be drawn at baseline, at 12 weeks’ follow-up, and at 1 month after childbirth. Samples will be stored in separate freezers at−80 °C until analysis. All samples will be analyzed on the same day and under the same conditions by a biochemist blinded to the severity of depressive symptoms and participants’ diagnostic status.

### Other outcomes

Standard obstetric outcomes will be analyzed: gestational age, gestational diabetes mellitus, gestational hypertension or preeclampsia, induced labor, estimated blood loss, cesarean section, operative vaginal delivery, birthweight, 1-min and 5-min Apgar scores, and neonatal intensive care unit admission. Data will be collected on discontinuation of the allocated interventions.

### Participant protection and reporting of adverse events

We define an adverse event as any unwanted or unintended sign, symptom, or disease observed in the trial participants, whether or not it is caused by study intervention. Safety issues will be monitored by interview, telephone, or email. Researchers and clinical research coordinators will record and evaluate any suspected adverse events. The follow criteria will guide evaluation of the severity of adverse [34].

1. Mild: Some symptoms or signs observed. Treatment is not required for the participant to continue in the trial.
2. Moderate: Some symptoms or signs observed. The participant may reduce the dose of test capsules or receive treatment such as additional drugs to continue the trial.
3. Severe: Clinical symptoms are severe enough to interfere with daily activities. Discontinuation of test capsules may be necessary.

The following are always regarded as serious adverse events, regardless of test dosage.

1. An event that results in death
2. A life-threatening event
3. An event that requires hospitalization (or extends an existing hospitalization period) in order to provide treatment
4. An event that results in a permanent or prominent disorder or a failure in body function
5. An event that results in a subsequent congenital anomaly or deficiency
6. Other events considered medically significant

Any serious adverse event will be reported to and assessed by the Data and Safety Monitoring Board as soon as possible. The ethics committee will also receive the report. Other adverse events will be reported and evaluated regularly. Information on concomitant drug use and reason for use will also be reported.

### Estimation of sample size

The expected difference in total HAMD score between the two arms at 12 weeks’ follow-up is set at 4.7 according to a previous study [28]. It is expected that 12 % of participants will discontinue the test capsules or drop out of the trial. Given an α level of 0.05 (two-tailed), a β level of 0.10, and standard deviation of 5, the desired number of participants will be 28 in each arm and 56 in total. Because a previous study suggested that seafood consumption varied across countries [33] and this may have a major effect on the efficacy of omega-3 PUFAs, 56 participants will be recruited at each site in Japan and Taiwan. Thus, the total number of participants will be 112. However, it is possible that enrollment will be halted before reaching this target if significant financial or logistical factors arise, or that number of cases at one of the sites may exceed the target number depending on the accumulation of cases at the other site.

### Statistical analysis

#### Primary analysis

Primary analysis will be conducted according to the intention-to-treat principle. Mean score differences, 95 % confidence intervals, and P values will be calculated using analysis of covariance in order to examine whether participants receiving omega-3 PUFAs supplementation score on average 4.7 points lower on the HAMD at 12 weeks’ follow-up than participants receiving placebo. Two-tailed tests will be used, with the α level set at 5 %. The assignment remains masked, but any background factors that may predict primary outcome will be entered as covariates if such factors are found.

#### Exploratory analysis

We will conduct exploratory analysis of secondary outcomes and sub-groups and therefore multiplicity will not be controlled. A mixed effect model with repeated measures will be evaluated by regression. The validity of the findings will be examined through sensitivity analysis, including per protocol analysis and analysis of the method used to impute missing variables.

### Ethical considerations

This study protects the rights and welfare of participants in the spirit of ethical guidelines outlined under the Declaration of Helsinki. Informed consent to participate in the study will be obtained from all participants. Personal information about potential and enrolled participants will be strictly secured to avoid external leaks before, during, and after the trial. No special compensation will be paid in the event of health damage directly related to the research. The research plan was deliberated on and approved by the following ethics committees: Tokyo Medical University, Japan, 26 June 2013; and China Medical University, Taiwan, 6 December 2013. If the protocol needs to be changed for any reason, the principal investigators will communicate this with the institutional review boards.

### Data and safety monitoring

The data manager will use a premade template to create a report to be sent to the principal investigator and the Data and Safety Monitoring Board once every 6–12 months. The members of the Data and Safety Monitoring Board are Professor Koichiro Watanabe (psychopharmacology), Dr. Katsumi Ikeshita (clinical psychiatrist), and Dr. Tomohiro Nakao (psychotherapy), who are professionals with clinical trial experience with no involvement in the trial. The monitoring report includes the following.

1. Progress of the study and case registration
2. Psychiatric evaluation status and any relevant issues
3. Any adverse events
4. Discussion of other relevant issues

### Discontinuing intervention

Intervention will be discontinued if the participant does not wish to continue taking the trial substance or placebo, or if continuing the intervention would be difficult because medically required concomitant medication may be affected by the trial substance. Subsequent evaluations and follow-up will proceed as designed.

### Presentation of the trial results

The findings of the trial will be presented in medical journals and at academic conferences. If particularly noteworthy results are obtain or the study is accepted by an influential academic publication, an application for a press release will be made. The principal investigators are, in general, listed as corresponding authors. The order of presentation of the first author and co-authors will be determined according to their intellectual contributions. Individuals who join the study after its approval and work toward implementing and conducting the study may obtain authorship pending approval by the principal investigators.

## Discussion

The findings of the SYNCHRO study are expected to influence decisions on the clinical care of pregnant women with depressive symptoms. The strengths of the study include its double-blind, placebo-controlled design, its multicenter, multi-country collaboration, and its evaluation of an EPA-rich supplement.

Maternal depression can have a significant harmful influence on both mothers and children. Due to the possibility of adverse effects of antidepressants, omega-3 PUFAs may provide a safe alternative for pregnant women with depressive symptoms. So far, only 2 RCTs have examined the efficacy of omega-3 PUFAs with a high ratio of EPA for depression in pregnancy [28, 43]; one of which recruited pregnant women without current depression but with a history of depression [43]. In addition, the result of one study on omega-3 PUFAs conducted in one country may not be able to be applicable to other countries due to differences in fish consumption [33]. The SYNCHRO study should provide evidence for two different populations in Asia and contribute to promoting mental health of large numbers of mothers and children.

## Trial status

This randomized trial is now enrolling participants and conducting follow-up.

## Additional file

Additional file 1: SPIRIT Checklist. (DOC 124 kb)

## Abbreviations

BDI-II: Beck depression inventory II
BDNF: Brain-derived neurotrophic factor
CBT: Cognitive behavioral therapy
DHA: Docosahexaenoic acid
EPA: Eicosapentaenoic acid
EPDS: Edinburgh postnatal depression scale
HAMD: Hamilton rating scale for depression
IPT: Interpersonal psychotherapy
MDD: Major depressive disorders
MINI: Mini international neuropsychiatric interview
PUFA: Omega-3 polyunsaturated fatty acid
RCT: Randomized controlled trial
